# Hepatitis C treatment outcomes for Australian First Nations Peoples: equivalent SVR rate but higher rates of loss to follow-up

**DOI:** 10.1186/s12876-022-02416-5

**Published:** 2022-07-11

**Authors:** Paul J. Clark, Patricia C. Valery, James Ward, Simone I. Strasser, Martin Weltman, Alexander Thompson, Miriam T. Levy, Barbara Leggett, Amany Zekry, Julian Rong, Peter Angus, Jacob George, Steven Bollipo, Bruce McGarity, William Sievert, Gerry Macquillan, Edmund Tse, Amanda Nicoll, Amanda Wade, Geoff Chu, Damian Harding, Wendy Cheng, Geoff Farrell, Stuart K. Roberts

**Affiliations:** 1 Department of Gastroenterology, Mater Hospital Brisbane, Raymond Terrace, South Brisbane, QLD 4101 Australia; 2 Department of Gastroenterology, Princess Alexandra Hospital, Alcohol and Drug Assessment Unit, Inala Indigenous Health Centre and Faculty of Medicine, The University of Queensland, QIMR Berghofer Medical Research Institute, Brisbane, QLD Australia; 3 QIMR Berghofer Medical Research Institute, Faculty of Medicine, The University of Queensland, Brisbane, QLD Australia; 4 UQ Poche Centre for Indigenous Health, Faculty of Health and Behavioural Sciences, The University of Queensland, Brisbane, QLD Australia; 5 AW Morrow Gastroenterology and Liver Centre Royal Prince Alfred Hospital, University of Sydney, Sydney, NSW Australia; 6 Hepatology Services, Nepean Hospital, Penrith, NSW Australia; 7 Department of Gastroenterology, St Vincent’s Hospital, Melbourne, VIC Australia; 8 Department of Gastroenterology and Liver, Liverpool Hospital, Sydney, NSW Australia; 9 Department of Gastroenterology and Hepatology, Royal Brisbane and Women’s Hospital, Brisbane, QLD Australia; 10 Department of Gastroenterology and Hepatology, St George Hospital, Sydney, NSW Australia; 11 Gippsland Gastroenterology, Latrobe Regional Hospital, Traralgon, VIC 3844 Australia; 12 Department of Gastroenterology and Hepatology, Austin Hospital, Melbourne, VIC Australia; 13 Storr Liver Unit, Westmead Hospital, Westmead, NSW Australia; 14 Gastroenterology Department, John Hunter Hospital, New Lambton, NSW Australia; 15 Bathurst Liver Clinic Bathurst Hospital, Bathurst, NSW Australia; 16 Gastrointestinal and Liver Unit, Monash Health, Melbourne, VIC Australia; 17 Liver Transplant Unit Sir Charles Gairdner Hospital, Nedlands Perth, WA Australia; 18 Hepatology, Royal Adelaide Hospital, Adelaide, SA Australia; 19 Eastern Health Box, Hill, VIC Australia; 20 Barwon Health Liver Clinic University Hospital, Geelong, VIC Australia; 21 Orange Liver Clinic, Orange Hospital, Orange, NSW Australia; 22 Department of Gastroenterology and Hepatology, Lyell McEwin Hospital, Vale, SA Australia; 23 Department of Gastroenterology and Hepatology, Royal Perth Hospital, Perth, WA Australia; 24 Gastroenterology and Hepatology Unit Canberra Hospital, Canberra, ACT Australia; 25 The Alfred Hospital, Melbourne, VIC Australia

**Keywords:** Sustained viral response, Liver fibrosis, Data linkage, Loss to follow-up

## Abstract

**Background:**

First Nations Peoples of Australia are disproportionally affected by hepatitis C (HCV) infection. Through a prospective study we evaluated the outcome of direct-acting antiviral (DAA) therapy among First Nations Peoples with HCV infection.

**Methods:**

Adults who initiated DAA therapy at one of 26 hospitals across Australia, 2016–2019 were included in the study. Clinical data were obtained from medical records and the Pharmaceutical and Medicare Benefits Schemes. Outcomes included sustained virologic response (SVR) and loss to follow-up (LTFU). A multivariable analysis assessed factors associated with LTFU.

**Results:**

Compared to non-Indigenous Australians (n = 3206), First Nations Peoples (n = 89) were younger (*p* < 0.001), morel likely to reside in most disadvantaged (*p* = 0.002) and in regional/remote areas (*p* < 0.001), and had similar liver disease severity. Medicines for mental health conditions were most commonly dispensed among First Nations Peoples (55.2% vs. 42.8%; *p* = 0.022). Of 2910 patients with follow-up data, both groups had high SVR rates (95.3% of First Nations Peoples vs. 93.2% of non-Indigenous patients; *p* = 0.51) and ‘good’ adherence (90.0% vs. 86.9%, respectively; *p* = 0.43). However, 28.1% of First Nations Peoples were LTFU vs. 11.2% of non-Indigenous patients (*p* < 0.001). Among First Nations Peoples, younger age (adj-OR = 0.93, 95% CI 0.87–0.99) and treatment initiation in 2018–2019 vs. 2016 (adj-OR = 5.14, 95% CI 1.23–21.36) predicted LTFU, while higher fibrosis score was associated with better engagement in HCV care (adj-OR = 0.71, 95% CI 0.50–0.99).

**Conclusions:**

Our data showed that First Nations Peoples have an equivalent HCV cure rate, but higher rates of LTFU. Better strategies to increase engagement of First Nations Peoples with HCV care are needed.

**Supplementary Information:**

The online version contains supplementary material available at 10.1186/s12876-022-02416-5.

## Background

Aboriginal and Torres Strait Islander peoples, the First Nations Peoples of Australia, are disproportionally affected by hepatitis C (HCV) infection [1], with rates of diagnosis between 3 and 5 times higher than non-Indigenous Australians. Of concern is rates for HCV diagnosis in First Nations Peoples under the age of 25 years where rates of diagnosis in the period 2016–2019 were between 6 and 8 times greater than same aged non-Indigenous Australians.

Universal access to direct-acting antiviral (DAA) therapy became available for all Australians via the Pharmaceutical Benefits Scheme (PBS) in 2016, aiming to ensure accessibility to cure (Sustained Viral Response, SVR). Yet access to cure is not truly universal. Many barriers undermine Australia’s pursuit of HCV elimination.

DAA access was associated with a reduction in HCV notification rate for non‑Indigenous Australians from 42.0 to 33.4 per 100,000 between 2015 and 2018 [1]. During this same period, notification rates for First Nations Peoples already 4–5 times higher than non-Indigenous Australians, barely reduced at all (from 174.0 to 163.6 per 100,000) [1]. Data on HCV treatment uptake and SVR are scant for First Nations Peoples. During 2014–2020, approximately 93,130 individuals initiated DAA treatment through the PBS [2]. At a population level, coincident with DAA availability has been a reduction in HCV-related decompensated cirrhosis (21%) and liver-related deaths (17%), and a plateauing of hepatocellular carcinoma (HCC) rates [3]. With limited evidence for reduction in HCV notifications for First Nations Peoples, these reductions in HCV-liver related morbidity and mortality are unlikely to translate to this patient group. First Nations Peoples are at increased risk of cirrhosis and poorer outcomes with cirrhosis, such as hospitalisation and HCC [4]. Optimising opportunities for HCV cure is critical to prevent the morbidity and mortality from HCV-related liver disease.

First Nations Peoples with HCV may not experience HCV care in the same way as non-Indigenous Australians [5]. We evaluated outcomes among First Nations Peoples with HCV in a real-world setting to better understand how HCV treatment programs may better adapt to the needs of this population. More specifically, we assessed severity of liver disease at initiation of HCV treatment, SVR, and rate of loss to follow-up (LTFU). We also explored factors associated with LTFU among First Nations Peoples.

## Methods

Details of the OPERA-C study have been described previously [6]. Briefly, the OPERA-C study included Australian adults with HCV who initiated DAA therapy at one of 26 hospitals across Australia during Feb-2016 to Dec-2019. The decision for antiviral treatment initiation and the specific DAA treatment were determined by local clinicians following Australian guidelines, considering HCV genotype, cirrhosis and comorbidities. With informed patient consent, a study nurse collected the patient details about DAA initiation and at 6 monthly intervals for 2 years. Data linkage was undertaken to Medicare Benefits Schedule (MBS) and PBS.

### Data collection and governance

Sociodemographic and clinical data were obtained from medical records. Residential postcode was used to categorise area-level rurality [7] and socio-economic status using the Index of Relative Socioeconomic Advantage and Disadvantage [8]. Clinical data included HCV diagnosis, HCV treatment and transmission history, concurrent opioid replacement therapy, risk factors for liver disease including significant alcohol intake (≥ 40 g of ethanol per day), and comorbidities. Follow-up data collected every six months included HCV diagnosis (e.g. reinfection), HCV treatment, and treatment response.

Liver fibrosis was assessed using transient elastography (TE) and Fibrosis-4 (FIB-4) [9] scores. Pre-defined risk thresholds for cirrhosis for transient elastography [10] and FIB-4 test were used [9]. TE score < 8 kiloPascals (kPa) was considered as ‘no/minimal liver fibrosis’, 8–12.5 kPa ‘moderate/advanced fibrosis’, and > 12.5 kPa was considered cirrhotic. FIB-4 test cut-off value of > 3.25 was categorized as advanced liver fibrosis (positive predictive value 65%; specificity of 97%) [9]. Cirrhosis compensation was classified using Child–Pugh and MELD scores.

SVR was defined as undetectable viral load at least 12 weeks after completion of DAA therapy. Patients who did not attend clinic for SVR testing 52 weeks or more after enrolment in OPERA-C and had not died or been discharged from clinic were classified as LTFU. The 52 week cut-off allowed better capture of real-world care where patients may have late SVR testing.

Data were linked to Commonwealth PBS and MBS records. Complete dispensing and MBS histories from May-2015 to Sep-2019 were extracted. We selected medication dispensing histories and service use 12-month prior to and during DAA therapy. Comorbid medical conditions were derived using the RxRisk-V [11] model which has been validated in the Australian setting [12, 13]. Outputs were reviewed by a hepatologist (PJC) and a pharmacist (KH) for confounding indications (e.g. propranolol for portal hypertension would not activate a hypertension diagnosis). Rx-Risk-V classification is available in Additional file 1: Table S1.

### Statistical analysis

Analyses were conducted using Stata/SE (Version 15; Stata Corporation, College Station, TX). Group comparisons used parametric and non-parametric methods. Multivariable analysis (MVA) using linear regression models assessed differences in fibrosis (log transformed FIB-4 to reduce skew) adjusting for age. The rate of medication dispensing was calculated using person days at risk as a denominator. Poisson regression compared medication dispensing rates according to Indigenous status (Wald tests), adjusting for age, reported as incidence rate ratios (IRR) with 95% confidence intervals (CI). Logistic MVA assessed factors associated with LTFU among First Nations Peoples. The final logistic multivariable model was determined based on the results of the bi-variable analysis but also taking into account our understanding of the relationships and dependencies among variables, their clinical relevance, our previous analysis of this cohort [6], and informed by a previous study on LTFU in HCV care population [16]. The final model included age, FIB-4 score, number of comorbidities assessed by the RxRisk, Year of HCV treatment initiation, and type of service.

## Results

**Fig. 1:**
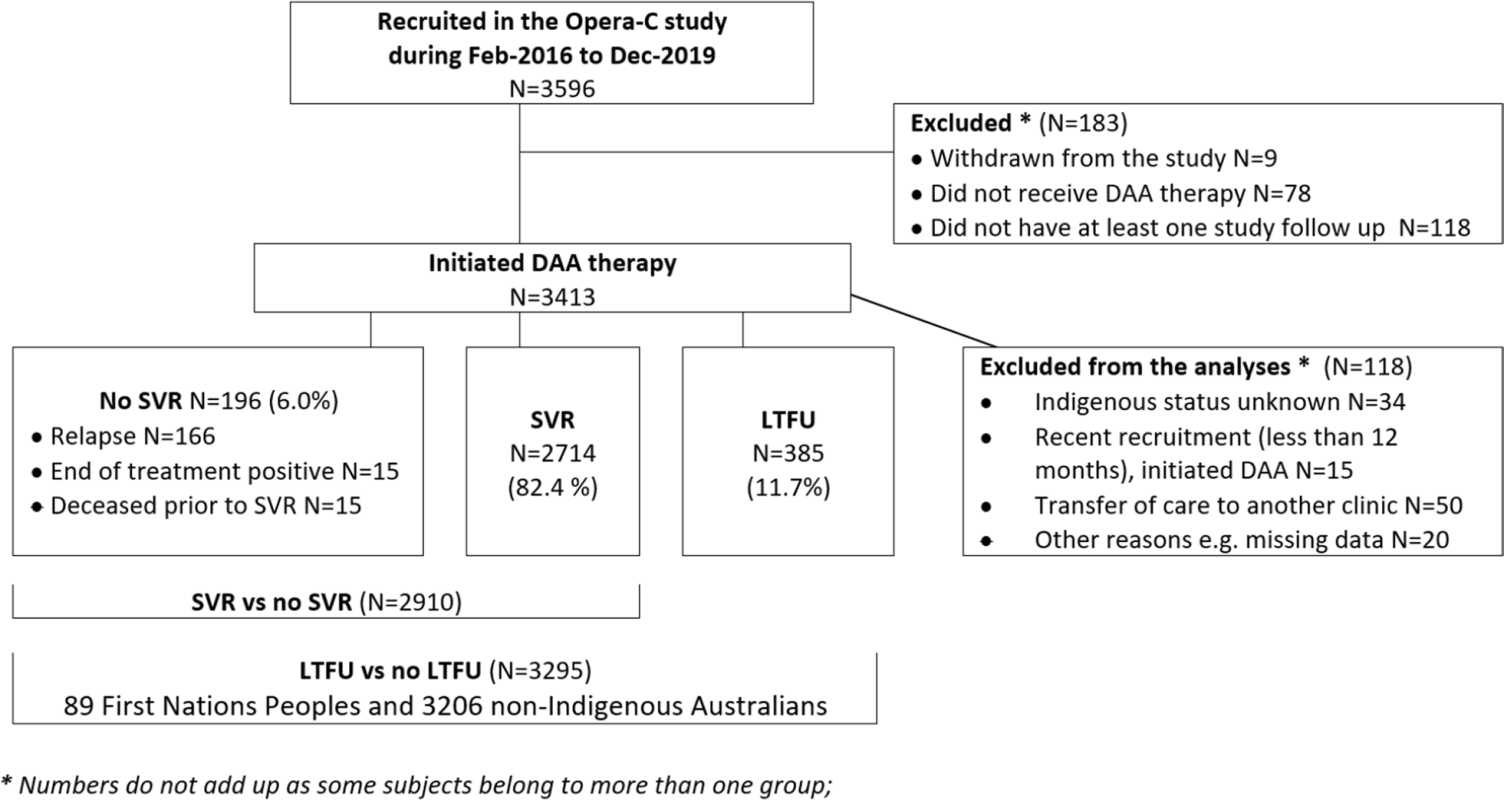
Flow chart for patient inclusion in the analyses of SVR and LTFU

Of 3295 patients enrolled in the OPERA-C study, 89 (2.7%) identified as First Nations Peoples (Fig. 1). Relative to non-Indigenous, First Nations Peoples were younger (48 years (SD = 10.5) vs. 52 years (SD = 10.4); *p* < 0.001), more lived in areas of relative social disadvantage (67.4% in the two most disadvantaged quintiles compared to 50.7%, *p* = 0.002), and more lived in regional/remote areas (43.8% vs. 25.3%, *p* < 0.001; Table 1). Diabetes was present in about 1-in-4 patients and did not differ based on Indigenous status (*p* = 0.70) nor did Hepatitis B or HIV infection. Indigenous patients had higher rates of alcohol abstinence compared to non-Indigenous (75.0% vs. 58.6%, *p* = 0.018).

**Table 1:** Patient demographic and clinical characteristics at recruitment for First Nations Peoples and non-Indigenous Australians

	First Nations Peoples	Non-Indigenous Australians	
	N = 89	N = 3206	*p* value
*Data source: patient medical records*			
Age (mean, SD)	48.1 (10.48)	52.1 (10.43)	< 0.001*
Gender			
Male	62 (69.7%)	2110 (65.8%)	0.45^‡^
Socioeconomic status			
Q1 most affluent/Q2/Q3	29 (32.6%)	1580 (49.3%)	0.002^‡^
Q4/Q5 most disadvantaged	60 (67.4%)	1623 (50.7%)	
Remoteness of residence			
Major city	50 (56.2%)	2393 (74.7%)	< 0.001^‡^
Regional/remote	39 (43.8%)	812 (25.3%)	
Diabetes	21 (23.6%)	808 (25.4%)	0.70^‡^
Hepatitis B surface antigen	2 (2.5%)	44 (1.7%)	0.65^¥^
Hepatitis B surface antibody	33 (42.3%)	1044 (44.2%)	0.75^‡^
Hepatitis B core antibody	26 (36.6%)	635 (31.5%)	0.37^‡^
HIV	1 (1.6%)	24 (1.3%)	0.58^¥^
Prescribed opioid substitute	21 (24.1%)	437 (14.4%)	0.012^‡^
Current alcohol consumption			
Zero alcohol	54 (75.0%)	1411 (58.6%)	0.018 ^¥^
< 40 g/day	14 (19.4%)	688 (28.6%)	
≥ 40 g/day	4 (5.6%)	307 (12.8%)	
Cirrhosis	32 (36.0%)	1023 (32.2%)	0.45^‡^
Liver fibrosis assessment			
FIB-4 score (median, IQR)^#^	1.36 (0.78–2.41)	1.63 (1.03–2.97)	0.03^€^
FIB-4^#^			
No liver fibrosis (FIB-4 ≤ 3.25)	68 (80.0%)	2173 (77.9%)	0.79^‡^
Liver fibrosis FIB-4 > 3.25	17 (20.0%)	616 (22.1%)	
Liver stiffness (kPa) (median, IQR)^†^	7.55 (5.30–13.70)	7.50 (5.50–13.10)	0.72 €
Liver stiffness groups^†^			
< 8.0 kPa (minimal fibrosis)	32 (53.3%)	1317 (54.7%)	0.78^‡^
8.0–12.5 kPa (moderate fibrosis)	10 (16.7%)	463 (19.2%)	
> 12.5 kPa (advanced fibrosis/cirrhosis)	18 (30.0%)	629 (26.1%)	
Hepatocellular carcinoma before DAA treatment	4 (4.5%)	62 (1.9%)	0.10^¥^
*Data source: MBS*^β^			
Mental health services	25 (28.1%)	771 (24.0%)	0.38^‡^
General Practitioner or Specialist (excluding psychiatrist)	86 (96.6%)	3012 (93.9%)	0.37^‡^
Number of visits (mean, SD)	14.4 (11.7)	11.8 (11.0)	0.029*
After hours services	18 (20.2%)	806 (25.1%)	0.29^‡^
Multidisciplinary care plan or case conferences	27 (30.3%)	965 (30.1%)	0.96^‡^
Addiction services	1 (1.1%)	4 (0.1%)	0.13^¥^
*Data source: PBS*^β,¶^			
Total number Rx-Risk-V comorbidities (median, IQR)	2 (1–5)	2 (1–4)	0.052^€^
0 (no Rx-Risk comorbidity)	12 (13.8%)	599 (19.8%)	0.058^‡^
1	17 (19.5%)	640 (21.1%)	
2	17 (19.5%)	514 (17.0%)	
3	6 (6.9%)	424 (14.0%)	
4	10 (11.5%)	308 (10.2%)	
5 or more Rx-Risk comorbidities	25 (28.7%)	542 (17.9%)	
RxRisk-V categories			
Pain (opioids)	37 (42.5%)	1052 (34.8%)	0.13^‡^
Depression	36 (41.4%)	843 (27.8%)	0.006^‡^
Gastric acid disorders	24 (27.6%)	782 (25.8%)	0.71^‡^
Psychotic illness	23 (26.4%)	402 (13.3%)	< 0.001^‡^
Anxiety and tension	20 (23.0%)	650 (21.5%)	0.73^‡^
Reactive airways disease	23 (26.4%)	587 (19.4%)	0.10^‡^
Smoking cessation medication	17 (19.5%)	268 (8.9%)	< 0.001^‡^
Congestive heart failure—hypertension	3 (3.4%)	26 (0.9%)	0.046^¥^
Selected RxRisk-V categories grouped			
Cardiovascular disease**	18 (20.7%)	554 (18.3%)	0.57^‡^
Mental health^∞^	48 (55.2%)	1296 (42.8%)	0.022^‡^

Data are presented as n (%) unless specified

^#^Missing data for 4 First Nations Peoples and 307 non-Indigenous Australians

^†^Missing data for 29 First Nations Peoples and 797 non-Indigenous Australians

^β^Service use or medication dispensing at least once in the 12 months prior to DAA therapy unless specified otherwise

^¶^PBS data not available for 2 First Nations Peoples and 179 non-Indigenous Australians

^*^Student’s *t* test

^‡^Pearson's chi-squared

^¥^Fisher's exact test

^€^Wilcoxon rank-sum

^**^Included four RxRisk categories namely congestive heart failure—hypertension, hypertension, ischaemic heart disease—angina, and ischaemic heart disease—hypertension

^**∞**^Included four RxRisk categories namely bipolar, depression, psychotic illness, and anxiety

There was no difference in liver fibrosis between the two groups (median FIB-4, *p* = 0.802) or cirrhosis (about one-third of all patients having cirrhosis, *p* = 0.45; adjusting for age *p* = 0.08). The proportion of compensated cirrhosis was similar, (prevalence Child–Pugh class A 27.8% for First Nations Peoples vs. 28.6% for non-Indigenous; *p* = 0.93).

Adjusting for age, First Nations Peoples had a higher rate of comorbid conditions compared to non-Indigenous Australians (adjusted IRR = 1.26 95% CI 1.11–1.42; *p* < 0.001). PBS data showed that the group of medicines for mental health conditions namely anxiety, depression, bipolar and psychotic illnesses were more commonly used in First Nations Peoples (55.2%) and prescribed more often than for non-Indigenous Australians (42.8%; *p* = 0.022). Opioid analgesia was the most commonly dispensed individual medication category among both patient groups (42.5% of First Nations Peoples; *p* = 0.13). The list of most common medication groups (> 20%) among First Nations Peoples or where significant according to Indigenous status is described in Table 1 (full list in Additional file 1: Table S2).

Regarding the use of health professional services included in the MBS, data showed that First Nations Peoples had a higher number of general practitioner (GP) or specialist visits than non-Indigenous Australians (mean number of visits 14.4 [SD = 11.7] vs. 11.8 [SD = 11.0], respectively; *p* = 0.029). MBS data for selected services showed that ever versus never use in the 12 months prior to DAA therapy was similar between the two groups (e.g. mental health services *p* = 0.38, addiction services *p* = 0.13).

### HCV assessment, treatment and engagement

The two groups were comparable with regards to the most common genotypes (G1 and G3; *p* = 0.19), duration of HCV infection (*p* = 0.83), viral load (*p* = 0.14), and the most common mode of HCV acquisition (injection drug use (IDU); *p* = 0.11; Table 2). A higher proportion of First Nations Peoples was prescribed opioid substitution therapy (24.1% vs. 14.4%, *p* = 0.012), and had tattoo as the attributed mode of HCV acquisition (30.3% vs. 16.4%, *p* < 0.001) compared to non-Indigenous patients. Regarding HCV treatment, fewer First Nations Peoples had prior HCV treatment (10.1% vs. 20.3%, *p* = 0.018), but this was not significant after adjustment for age (*p* = 0.07). There was no significant difference in DAA regimen prescribed (*p* = 0.11). Treating clinician’s assessment of patient adherence was ‘good’ for most patients with SVR determination and did not differ by Indigenous status (90.0% of First Nations Peoples vs. 86.9% of non-Indigenous patients, *p* = 0.43).

**Table 2:** HCV assessment at recruitment and treatment according for First Nations Peoples and non-Indigenous Australians

	First Nations Peoples	Non-Indigenous Australians	
	N = 89	N = 3206	*p* value
Genotype			
G1	49 (55.1%)	1723 (53.7%)	0.19^‡^
G3	28 (31.5%)	1208 (37.7%)	
Other	12 (13.5%)	275 (8.6%)	
Duration of HCV infection in years (mean, SD)^a^	20.75 (13.13)	22.80 (11.96)	0.14
Viral load IU/ml^b^ (median, IQR)	1,220,000 (281,000–4,230,000)	1,258,925 (351,000–3,740,000)	0.83^€^
Mode of HCV Acquisition^c^			
Injection drug use	67 (75.3%)	2152 (67.2%)	0.11^‡^
Tattoo	27 (30.3%)	525 (16.4%)	< 0.001^‡^
Blood transfusion	8 (9.0%)	284 (8.9%)	0.97^‡^
HCV treatment prior to DAA-era	9 (10.1%)	651 (20.3%)	0.018^‡^
Regimen			
PEGIFN/IFN ± RBV	8 (88.9%)	486 (74.7%)	1.00^¥^
1st generation PEGIFN/protease inhibitors	1 (11.1%)	86 (13.2%)	
DAA ± RBV	0 (0.0%)	19 (2.9%)	
RCT not brought forward or not otherwise specified	0 (0.0%)	60 (9.2%)	
Treatment response			
SVR (presumed re-infection)	0 (0.0%)	8 (1.2%)	0.55^¥^
Relapse	2 (22.2%)	257 (39.5%)	
Non-responder	6 (66.7%)	313 (48.1%)	
Unknown	1 (11.1%)	73 (11.2%)	
HCV DAA therapy regimen			
Sofosbuvir/Ledipasvir	28 (31.5%)	1276 (39.8%)	0.13^¥^
Sofosbuvir + Daclastavir	22 (24.7%)	926 (28.9%)	
Sofosbuvir/Velpatasvir	19 (21.3%)	557 (17.4%)	
Sofosbuvir + Ribavirin	3 (3.4%)	93 (2.9%)	
Elbasvir/Grazoprevir	9 (10.1%)	165 (5.1%)	
Glecaprevir/Pibrentasvir	7 (7.9%)	107 (3.3%)	
Ombitasvir/Paritaprevir/Ritonavir/Dasabuvir	1 (1.1%)	61 (1.9%)	
Sofosbuvir/Velpatasvir/Voxilaprevir	0 (0.0%)	5 (0.2%)	
Miscellaneous DAAs	0 (0.0%)	16 (0.5%)	
Treatment included ribavirin^d^	1 (1.1%)	86 (2.7%)	0.73^¥^
Lost to follow-up			
No LTFU	64 (71.9%)	2846 (88.8%)	< 0.001^‡^
LTFU	25 (28.1%)	360 (11.2%)	

Data are presented as n (%) unless specified

^a^Missing data for 12 First Nations Peoples and 383 non-Indigenous Australians

^b^Missing data for 22 First Nations Peoples and 547 non-Indigenous Australians

^c^Patients may belong to more than one group unless specified

^d^Excluding Sofosbuvir + Ribavirin

^‡^Pearson's chi-squared

^¥^Fisher's exact test

^€^Wilcoxon rank-sum

^¶^Student’s *t* test

For 2910 individuals with SVR results available, SVR was equivalent among First Nations Peoples (n = 64, 95.3%) and non-Indigenous (n = 2653, 93.2%; *p* = 0.51). However, higher rates of LTFU for SVR testing in First Nations Peoples was observed (28.1% vs. 11.2%, respectively; *p* < 0.001).

Analysis restricted to First Nations Peoples showed that, compared to patients who engaged in HCV care, patients who were LTFU were significantly younger (*p* = 0.001), had shorter duration of HCV infection (*p* = 0.020), less liver fibrosis (*p* = 0.019), less cirrhosis (*p* = 0.019), and a higher proportion were late-initiators of HCV treatment (2018–2019 vs. 2016 (*p* = 0.014). In MVA, LTFU was higher among younger patients (adj-OR = 0.93, 95% CI 0.87–0.99; *p* = 0.026) and those who initiated HCV treatment in 2018–2019 vs. 2016 (adj-OR = 5.14, 95% CI 1.23–21.36; *p* = 0.025), while patients with more liver fibrosis had better engagement in HCV care (adj-OR = 0.71, 95% CI 0.50–0.99; *p* = 0.047). Details about these analyses are available in Additional file 1: Table S3.

## Discussion

In this study, antiviral treatment was just as effective for First Nations Peoples with HCV as for non-Indigenous Australians with these real-world Australian data consistent with clinical trials and other treatment settings [14–16]. In 2017, HCV notification rate in the First Nations Peoples in 5 Australian jurisdictions was 4.4 times higher than that of non-Indigenous Australians (168.1 per 100 000 vs. 38.4 per 100,000, respectively), and they had lower lifetime (37% vs. 47%) uptake of treatment [17]. In our study, once commenced DAA treatment adherence was high. Many patients with HCV have complex health needs and challenging social situations, however relative to non-Indigenous Australians with HCV, First Nations Peoples experienced higher rates of social disadvantage, more co-morbidity including psychiatric illness [18]. These competing social and medical needs constitute barriers to HCV cure for this population [4, 19].

In a recent Australian HCV study (REACH-C) [16], younger age was independently associated with LTFU in a cohort of Australians treated with DAA (adj-OR = 0.97, 95% CI 0.97–0.98, *p* < 0.01). Other predictors of LTFU were IDU and initiation of treatment after 2016, while HIV coinfection and previous interferon-based HCV treatment were associated with decrease in LTFU. Unlike our study, REACH-C had lower rates of liver disease, and in MVA no difference was seen in LTFU based on Indigenous identification. In our study all patients initiated HCV treatment through hospital services, while in the REACH-C study 53% of patients initiated HCV treatment through specialist liver clinics and 47% through other services (e.g. general practice, community health clinics, sexual health and drug and alcohol services, and prison). Hospital-based care is more difficult for First Nations Peoples to attend regularly, and there are opportunities to address barriers already cited such as racism in health care [20], structural barriers to access care (e.g. access to a suitable transport service) [21, 22], and communication between First Nations Peoples and health professionals [20, 23]. In our study, LTFU was 2.5-fold higher among First Nations Peoples than non-Indigenous Australians, and in MVA restricted to First Nations Peoples, younger age, treatment initiation in 2018–2019 versus 2016, and those with less liver fibrosis were predictors of LTFU. It is likely that the characteristics of the HCV population initiating treatment has changed over time, maybe including more vulnerable groups and patients with recent HCV infection. Some apathy toward SVR testing may have developed over time following observation of consistently high cure rate coupled with the change in the guidelines now suggesting that SVR testing is optional. Understanding the sociodemographic and clinical characteristics of First Nations Peoples with HCV offers insights to optimise engagement [18].

In this study, First Nations Peoples seeking treatment were likely to be younger and have less liver fibrosis and cirrhosis. Despite being younger, First Nations Peoples with HCV more frequently consulted health carer providers 12-month prior to and during DAA therapy (GP visits and specialists) and had more comorbidities. The group of medications for mental health conditions were the most commonly used group of medicines dispensed to First Nations Peoples with HCV, and dispensed more commonly than to non-Indigenous Australians. For First Nations Peoples, determinants of mental health illness are multi-factorial including life circumstances related to culture and spirituality, family and community kinships, historical, social and economic factors, fear of mental health services, loss of cultural identity, and connection to traditional lands and communities [24, 25]. Nasir et al. reported that the rates of anxiety, substance abuse and alcohol misuse among First Nations Peoples was nearly seven times higher than the general Australian population [24], with half the mental illness among First Nations Peoples living on traditional lands [24]. Opportunities for contributing to Australia’s HCV elimination targets among First Nations Peoples lay within Indigenous primary care services where community led responses are best at juggling competing health and social priorities. However barriers that prevent clients and primary care providers delivering optimal HCV treatment and cure need to be addressed. Facilitators of HCV treatment previously cited include incentives for providing HCV treatment, good systems including patient provider rapport and targeted case finding should be implemented to assist the elimination targets for First Nations Peoples [26].

Rates of alcohol abstinence were higher among First Nations Peoples included in the study compared to non-Indigenous patients. According to the Australian Institute of Health and Welfare, in 2016 a higher proportion of First Nations Peoples abstained from drinking alcohol compared to non-Indigenous Australians [27]. Despite higher abstinence rates, community efforts to reduce of harmful alcohol consumption among Indigenous Australian communities is ongoing, and rates of harmful drinking and harms caused by alcohol such as cirrhosis and mental health problems, remain health priority for First Nations Peoples [27]. We also showed that, similar to non-Indigenous Australians, the most common mode of HCV acquisition for First Nations Peoples was IDU. Methamphetamine, heroin and methadone IDU pose an emerging threat for regional and urban Indigenous communities alike [28]. Compared to non-Indigenous Australians, proportionally more First Nations Peoples were prescribed opioid substitution, reflecting positive impacts on the dynamic epidemiology of substance dependence. Social disruption and intergenerational trauma are recognised contributors to alcohol and substance abuse and mental illness among First Nations Peoples [29, 30]. Incidence of HCV and substance dependence and IDU are interwoven. Reducing the HCV burden for First Nations Peoples by increasing HCV treatments, will be undermined by failure to co-manage the addictions driving transmission. Contextualising these impacts is critical to understand the barriers First Nations Peoples must negotiate to access HCV treatment.

Despite the overrepresentation of First Nations Peoples among patients with HCV and higher morbidity and mortality [4, 19], services specifically targeting care among this patient group are few. The Deadly Liver Mob Program [5], for example, is a peer-driven HCV health promotion program operated in co-located needle and syringe programs and sexual health clinics in New South Wales that shown to improve clinic attendance. Nesting HCV treatment within Indigenous primary health services offers a mechanism to increase diagnosis and treatment while concurrently attending to diverse competing needs such as mental health, income and housing insecurity [18]. While increased GP visits in First Nations Peoples may reflect the higher rates of comorbidity, they also identify critical opportunities to improve HCV screening, treatment uptake, and cure at the GP level. The SCALE-C study, currently being tested in selected Aboriginal Community Controlled Health Services (ACCHS) [31], will evaluate a test-and-treat model and long-term impacts on HCV prevalence and transmission.

This study reflects HCV treatment delivered through mainstream services as patients were treated in hospitals by liver specialists in regional and metropolitan settings. Moreover, it does not include HCV treatment delivered through ACCHS and Aboriginal Medical Services which have been identified as critical in this priority population [32]. Other than liver-related comorbidities and diabetes, the use of medications as a surrogate to identify people with comorbidities may underestimate comorbidity. The use of non-pharmacological approaches are used for some conditions. Despite these limitations, the RxRisk-V has been validated and used in Australian and international cohorts [12, 13]. While the OPERA-C study included a large number of patients recruited across Australia, the relatively small number of First Nations Peoples reflects under-treatment. This is a limitation of treatment programs and may limit generalizability to all First Nations Peoples with HCV. Some patients who were LTFU may have accessed HCV care elsewhere, but this data could not be captured in the study, and linkage data does not provide sufficient clinical granularity to assess. Lastly, unmeasured confounders and omitted variable bias (when a relevant independent variable is not included in the model) can lead to biased associations between exposure and outcome in observational studies, and therefore must be considered when interpreting our findings.

## Conclusions

HCV cure reduces the risk for liver disease and liver cancer [3]. Our data from mainstream liver specialist centres showed that First Nations Peoples have an equivalent HCV cure rate, but higher rates of LTFU compared to non-Indigenous Australians. Higher rate of LTFU, together with underrepresentation for treatment, may be the reasons for higher rates of cirrhosis and liver cancer in this group [4, 19]. HCV infection is typically asymptomatic until advanced liver disease evolves, and when faced with more pressing psychological, medical or social needs may be de-prioritized by patients and/or health care providers. Holistic primary care is the ideal environment to nest HCV treatment, where competing social and health needs for First Nations Peoples might be better contextualised and co-managed. Importantly, integrating screening and treatment for HCV in primary care is critical to reduce the mortality gap for First Nations Australians. Specific efforts are needed to ensure that subsidised HCV medications can be translated to truly universal access to HCV cure or all Australians.

## Supplementary Information

**Additional file 1.** **Supplementary Table S1.** List of Rx-Risk-V comorbidity categories with corresponding medicine groups and Anatomical Therapeutic Chemical (ATC), and ATC codes of medications dispensed to patients included in the Opera-C study. **Supplementary Table 2.** Prevalence of comorbidity categories included in the Rx-Risk-V according for First Nations Peoples and non-Indigenous Australians. **Supplementary Table 3.** Logistic regression analysis of factors associated with loss to follow-up among First Nations Peoples.

## Data Availability

The data that support the findings of this study contain potentially sensitive and/or identifying information that could compromise the privacy of the participants. Therefore data are not publicly available. Data may, however, be available from the authors upon reasonable request with approval from relevant ethics committees.

## Abbreviations

CI: Confidence intervals
DAAs: Direct-acting antivirals
FIB-4: Fibrosis-4 score
HCV: Hepatitis C virus
IRR: Incidence rate ratios
IDU: Injection drug use
kPa: KiloPascals
LTFU: Loss to follow-up
MBS: Medicare Benefits Schedule
ORs: Odds ratios
OPERA-C: Observational, Prospective Epidemiological Registry in Australia of HCV Liver Disease
PBS: Pharmaceutical Benefits Scheme
SVR: Sustained virologic response
