# Supplementary material for: Hepatitis C treatment outcomes for Australian First Nations Peoples: equivalent SVR rate but higher rates of loss to follow-up
Source: BMC Gastroenterol. 2022 Jul 11;22:339. doi: 10.1186/s12876-022-02416-5 (PMC9275019; doi:10.1186/s12876-022-02416-5)
Supplement: Supplementary file 1 — Additional file 1. Supplementary Table S1. List of Rx-Risk-V comorbidity categories with corresponding medicine groups and Anatomical Therapeutic Chemical (ATC), and ATC codes of medications dispensed to patients included in the Opera-C study. Supplementary Table 2. Prevalence of comorbidity categories included in the Rx-Risk-V according for First Nations Peoples and non-Indigenous Australians. Supplementary Table 3. Logistic regression analysis of factors associated with loss to follow-up among First Nations Peoples. [file 12876_2022_2416_MOESM1_ESM.docx]

**Supplementary Table S1.** List of Rx-Risk-V comorbidity categories with corresponding medicine groups and Anatomical Therapeutic Chemical (ATC), and ATC codes of medications dispensed to patients included in the Opera-C study

| **Rx-Risk-V class** | **Medication groups and/or examples** | **ATC / PBS codes**  **included in the Rx-Risk-V as**  **previously reported[12, 33]** | **ATC / PBS codes of medications**  **dispensed to patients included**  **in the Opera-C study** |
| --- | --- | --- | --- |
| **Alcohol dependence** | e.g. Naltrexone, Acamprosate | N07BB03-N07BB04, V03AA01, N07BB01 | N07BB03, N07BB04 |
| **Allergies** | Antiallergic agents for local treatment of nasal congestion and antihistamines for systemic use e.g. promethazine | R01AC01–R01AD60, R06AD02–R06AX27, R06AB04 | R06AD02 |
| **Anticoagulation** | Anticoagulants e.g. rivaroxaban, apixaban | B01AA03-B01AB06, B01AB09, B01AE06, B01AE07, B01AF01, B01AF02, B01AX05 | B01AA03, B01AB01, B01AB04, B01AB05, B01AE07, B01AF01, B01AF02 |
| **Antiplatelet agents** | Antiplatelets e.g. clopidogrel, aspirin | B01AC04-B01AC30 | B01AC04, B01AC06, B01AC07, B01AC22, B01AC24, B01AC30 |
| **Anxiety and tension** | Anxiolytics e.g. diazepam, oxazepam | N05BA01-N05BA12, N05BE01 | N05BA01, N05BA04, N05BA12 |
| **Arrhythmias** | Antiarrhythmics e.g. flecainide, digoxin | C01AA05, C01BA01-C01BD01, C07AA07 | C01AA05, C01BC04, C01BD, C01BD01 |
| **Benign prostatic hypertrophy** | Alpha-adrenoreceptor blocking and alpha-adrenoreceptor antagonists e.g. dutasteride/tamsul | G04CA01–G04CA99, G04CB01, G04CB02  (must be male) | G04CA52 |
| **Bipolar disorders** | Antimanic agent e.g. lithium | N05AN01, N06AX | N06AX |
| **Congestive heart failure -hypertension** | Loop diuretics (e.g. furosemide) AND, Angiotensin converting enzyme (ACE) inhibitors (e.g. ramipril) or Angiotensin receptor blockers (ARB; e.g. irbesartan); Beta blockers selective for heart failure (e.g. carvedilol, nebivolol); Aldosterone antagonists specific for heart failure (e.g. eplerenone ) ^a, b, e, f^ | C03CA01-C03CC01, C09AA01-C09AX99,  C09CA01-C09CX99, C03DA04, C07AB07, C07AG02, C07AB12, C01EB17, C03DA01 | R03AC02, R03AC03, R03AC12, R03AC13, R03AC18, R03AK06, R03AK07, R03AK10, R03AK11, R03AL03, R03AL04, R03AL05, R03AL06, R03AL08, R03BA01, R03BA02, R03BA05, R03BA08, R03BB01, R03BB04, R03BB05, R03BB06, R03BB07, R03BC01, R03BC03, R03CC02, R03DA04, C03DA01 |
| **Dementia** | Anticholinesterases or other dementia medicines e.g. donepezil | N06DA02-N06DA04, N06DX01 | N06DA02, N06DX01 |
| **Depression** | Antidepressants e.g. mirtazapine, escitalopram | N06AA01-N06AG02, N06AX03-N06AX11,  N06AX13-N06AX18, N06AX21-N06AX23  (excluding amitriptyline) | N06AA02, N06AA04, N06AA10, N06AA12, N06AA16, N06AB03, N06AB04, N06AB05, N06AB06, N06AB08, N06AB10, N06AG02, N06AX03, N06AX11, N06AX16, N06AX18, N06AX21, N06AX23 |
| **Diabetes** | Insulins and oral hypoglycaemics e.g. glicazide, metformin, dapagliflozin | A10AA01-A10BH05, A10BX04, A10BX09, A10BX12, A10BH51 (A10BH03 also for hyperlipidaemia) | A10AB01, A10AB04, A10AB05, A10AB06, A10AC01, A10AD, A10AD01, A10AD04, A10AD06, A10AE04, A10AE05, A10BA02, A10BB01, A10BB07, A10BB09, A10BB12, A10BD02, A10BD07, A10BD08, A10BD10, A10BD11, A10BD13, A10BD15, A10BD19, A10BD20, A10BD21, A10BF01, A10BG03, A10BH01, A10BH02, A10BH03, A10BH04, A10BH05, A10BJ01, A10BJ05, A10BK01, A10BK03, A10BX11 |
| **Epilepsy** | Anticonvulsants e.g. levetiracetam, valproate | N03AA01-N03AX15, N03AX18, N03AX22 | N03AA03, N03AB02, N03AE01, N03AF01, N03AF02, N03AG01, N03AG04, N03AG06, N03AX03, N03AX09, N03AX11, N03AX12, N03AX14, N03AX15, N03AX18 |
| **Gastric acid disorder** | H2-antagonist (e.g. ranitidine) and proton pump inhibitors (e.g. esomeprazole) | A02BA01-A02BX05 | A02BA02, A02BA03, A02BA04, A02BC01, A02BC02, A02BC03, A02BC04, A02BC05, A02BD06, A02BX, A02BX02 |
| **Glaucoma** | Topical anti-glaucoma agents e.g. latanoprost | S01EA01-S01EB03, S01EC03-S01EX99, S01EA, S01EC, S01EC01 | S01EA, S01EA03, S01EA05, S01EB01, S01EC, S01EC01, S01EC03, S01EC04, S01EC54, S01ED01, S01EE, S01EE01, S01EE03, S01EE04, S01EE05 |
| **Gout** | Antigout agents e.g. allopurinol | M04AA01-M04AC01 | M04AA01, M04AA03, M04AB01, M04AC01 |
| **Hepatitis B** | Anti-virals for hepatitis B e.g. entecavir, lamivudine, tenofovir | J05AF08, J05AF10, J05AF11, J05AF05, J05AF07. Excluding PBS items for HIV | J05AF05, J05AF07, J05AF10 |
| **Hepatitis C** | Interferon and ribavirin combinations, protease inhibitors (e.g. glecaprevir), and direct acting antivirals for hepatitis (e.g. sofosbuvir/ledispavir) | J05AB54, J05AE11-J05AE12, L03AB10, L03AB11, L03AB60, L03AB61, J05AE14, J05AX14, J04AX15, J05AX65, J05AB04, J05AP01-J05AP58 | Not included in the RxRisk as hepatitis C is the disease of interest in the OPERA-C study |
| **HIV** | Anti-retrovirals e.g. dolutegravir, raltegravir | J05AG05, J05AX12, J05AE01-J05AE10, J05AR01-J05AR99, J05AX07-J05AX09,  J05AF01-J05AG04 (excluding ATC codes for hepatitis B) | J05AE03 , J05AE08, J05AE10, J05AF05, J05AG01, J05AR, J05AR03, J05AR06, J05AR08, J05AR13, J05AR17, J05AR18, J05AR19, J05AR20, J05AX08, J05AX12 |
| **Hyperkalaemia** | e.g. Sodium polystyrene sulfonate | V03AE01 | - |
| **Hyperlipidaemia** | Anti-lipaemic agents e.g. atorvastatin | C10AA01-C10BX09, A10BH03 (also for diabetes). Excluding C10AC01 (colestyramine) | A10BH03, C10AA01, C10AA03, C10AA04, C10AA05, C10AA07, C10AB04, C10AB05, C10AX09, C10AX13, C10BA02, C10BA05, C10BA06, C10BX03 |
| **Hypertension** | Thiazides, potassium-sparing agents, combination anti-hypertensives, other antihypertensives **^a, c^**  (e.g. candersartan, valsartan) | C03AA01-C03BA11, C03DB01-C03DB99,  C03EA01, C09BA02-C09BA09, C09DA02-C09DA08, C02AB01-C02AC05, C02DB02-C02KX02, C02KX04, C02CA01 | C02AB01, C02AC01, C02AC05, C02CA01, C02DB02, C03AA03, C03BA04, C03BA11, C03EA01, C09BA02, C09BA04, C09BA06, C09BA09, C09BB02, C09BB04, C09BB05, C09BB10, C09CA01, C09CA02, C09DA02, C09DA03, C09DA04, C09DA06, C09DA07, C09DA08, |
| **Hyperthyroidism** | Antithyroid therapy e.g. carbimazole | H03BA02, H03BB01 | H03BB01 |
| **Hypothyroidism** | Thyroid replacements e.g. levothyroxine | H03AA01-H03AA02 | H03AA01, H03AA02 |
| **Incontinence** | Antispasmodics specifically used in the urogenital tractus e.g. oxybutynin | G04BD01–G04BD99, G04BD | G04BD, G04CA52 |
| **Irritable bowel syndrome** | Specific drugs for inflammatory bowel syndrome, rectal antiinflammatories e.g. sulfasalazine | A07EC01-A07EC04, A07EA01-A07EA02,  A07EA06, L04AA33 | A07EA01, A07EC01, A07EC02 |
| **Ischaemic heart disease - angina** | Nitrates e.g. isosorbide mononitrate | C01DA02-C01DA14, C01DX16, C08EX02, C01DX | C01DA02, C01DA14, C01DX, C01DX16 |
| **Ischaemic heart disease - hypertension** | Beta-blockers, calcium channel blockers ^d^ e.g. amlopidine, metoprolol | C07AA01-C07AA06, C07AA8-C07AB01,  C07AG01, C08CA01-C08DB01, C09DB01-C09DB04, C09DX01, C09BB02-C09BB10,  C10BX03, C07AB03, C09DX03. Metoprolol requires testing according to PBS item code for indication | C07AA03, C07AB02, C07AB03, C07AG01, C08CA01, C08CA02, C08CA05, C08CA13, C09DB01, C09DB02, C09DB04, C09DX01, C09DX03, C10BX03 |
| **Liver failure** | e.g. lactulose and rifaximin | A06AD11, A07AA11 | A06AD11, A07AA11 |
| **Malignancies** | Antineoplastics agents (excluding topical and endocrine therapies) e.g. cyclophosphamide, sorafenid, carboplatin | L01AA01-L01XX41. Excluding immunosuppressants or immunomodulators used for other indications | L01AA01, L01AA09, L01BA01, L01BB02, L01BB03, L01BC01, L01BC02, L01BC05, L01BC06, L01CA02, L01CA04, L01CB01, L01CD01, L01CD02, L01DB01, L01DB03, L01DB06, L01XA01, L01XA02, L01XA03, L01XC02, L01XC03, L01XC06, L01XC07, L01XC12, L01XC17, L01XC18, L01XE01, L01XE02, L01XE05, L01XE18, L01XE27, L01XE29, L01XE42, L01XX05, L01XX19, L01XX27, L01XX32 |
| **Malnutrition** | Enteral nutritional supplements | B05BA01-B05BA10 | - |
| **Migraine** | Antimigraine medications e.g. pizotifen, sumatriptan | N02CA01-N02CX01 (excluding N02CX cyproheptadine) | N02CC01, N02CC02, N02CC03, N02CC04, N02CC06, N02CX01 |
| **Osteoporosis/Paget’s** | Bisphosphonates, selected drugs affecting bones e.g. denosumab | M05BA01-M05BB05, M05BX03, M05BX04, G03XC01, H05AA02, M05BX (raloxifene – must be women). Excluding PBS item code for hypercalcemia/multiple myeloma | M05BA03, M05BA04, M05BA07, M05BA08, M05BB02, M05BB03, M05BB04, M05BB05, M05BX04 |
| **Pain (opioids)** | Opiate containing medications e.g. oxycodone, tramadol | N02AA01-N02AX02, N02AX06, N02AX52, N02BE51 | N02AA01, N02AA03, N02AA05, N02AA55, N02AB03, N02AC, N02AE01, N02AJ06, N02AX02, N02AX06 |
| **Pain (inflammation)** | Nonsteroidal anti-inflammatory drugs (NSAIDS) e.g. celecoxib, meloxicam | M01AB01-M01AH06 | M01AB01, M01AB05, M01AC01, M01AC06, M01AE01, M01AE02, M01AE03, M01AG01, M01AH01 |
| **Pancreatic insufficiency** | Pancreatic exocrine enzyme replacement e.g. creon | A09AA02 | A09AA02 |
| **Parkinson’s disease** | Antiparkinson agents e.g. benzatropine | N04AA01-N04BX02 | N04AA01, N04AC01, N04BA02, N04BB01, N04BC05, N04BD02 |
| **Psoriasis** | Systemic and topical antipsoriatics e.g. calcipotriol | D05AA01-D05AA99, D05BB01-D05BB02,  D05AX02, D05AC01-D05AC51, D05AX52, D05AA | D05AA, D05AX02, D05AX52, D05BB02 |
| **Psychotic illness** | Antipsychotics e.g. olanzapine, quetiapine | N05AA1-N05AB02, N05AB06-N05AL07, N05AX07-N05AX13, N05AX16 | N05AA01, N05AB02, N05AC01, N05AD01, N05AE04, N05AE05, N05AF01, N05AF05, N05AH02, N05AH03, N05AH04, N05AH05, N05AL05, N05AX08, N05AX12, N05AX13, N05AX16 |
| **Pulmonary hypertension** | Antihypertensives for pulmonary arterial hypertension e.g. bosentan | C02KX01–C02KX05, PBS ITEM CODE 9547L, 9605M, C02KX | C02KX, C02KX01, C02KX04 |
| **Reactive airways diseases** | Inhaled bronchodilators e.g. salbutamon, tiotropium, fluticasone | R03AC02-R03DC03, R03DX05 | R03AC02, R03AC03, R03AC12, R03AC13, R03AC18, R03AK06, R03AK07, R03AK10, R03AK11, R03AL03, R03AL04, R03AL05, R03AL06, R03AL08, R03BA01, R03BA02, R03BA05, R03BA08, R03BB01, R03BB04, R03BB05, R03BB06, R03BB07, R03BC01, R03BC03, R03CC02, R03DA04 |
| **Renal disease (end stage)** | e.g. erythropoietin, calciferol, calcitriol, sevelamer, sucroferric oxyhydroxide | B03XA01-B03XA03, A11CC01-A11CC04,  V03AE02, V03AE03, V03AE05 | A11CC04, B03XA01, B03XA02, B03XA03, V03AE02, V03AE03, V03AE05 |
| **Smoking cessation** | e.g. nicotine, bupropion, varenicline | N07BA01-N07BA03, N06AX12, N07BA | N07BA, N07BA01, N07BA03 |
| **Steroid-responsive conditions** | Corticosteroids for systemic use e.g. prednisone, prednisolone | H02AB01-H02AB10 | H02AB01, H02AB02, H02AB04, H02AB06, H02AB07, H02AB08, H02AB09 |
| **Transplant** | Immune suppressants e.g. tacrolimus, mycophenolate | L04AA06, L04AA10, L04AA18, L04AD01, L04AD02 | L04AA06, L04AA10, L04AA18, L04AD01, L04AD02 |
| **Tuberculosis** | Antibiotics used in tuberculosis e.g. isoniazid | J04AC01-J04AC51, J04AM01-J04AM99 | J04AC01 |

**^a^** Medications such as captopril, valsartan, candesartan were coded as: a)'hypertension' if patients were not on beta blockers for congestive heart failure (e.g. metoprolol, bisoprolol, nebivolol) and not on a loop diuretic (e.g. furosemide); and as b) ‘congestive heart failure’ if patients were also on a beta-blocker for congestive heart failure and on a loop diuretic;

**^b^** Medications such as etacrynic acid were coded as: a)'hypertension' if patients were not on beta blockers for congestive heart failure (e.g. metoprolol, bisoprolol, nebivolol) and not ACE inhibitors or sartans; and as b) ‘congestive heart failure’ if patients were also on a beta-blocker for congestive heart failure and on ACE inhibitors or sartans;

^c^ Medications such as amiloride were coded as: a)'hypertension' if patients were not on beta blockers for congestive heart failure (e.g. metoprolol, bisoprolol, nebivolol); and as b) ‘congestive heart failure’ if patients were also on a beta-blocker for congestive heart failure;

^d^ Medications such as verapamil and diltiazem will be coded as: a) 'Ischemic heart disease' if patients are not on an anticoagulant; and as b) 'Arrhythmia' if patients are on an anticoagulant;

^e^ Spironolactone was coded as ‘congestive heart failure’ if patients were also on a beta-blocker for congestive heart failure and on ACE inhibitors or sartans;

^f^ Furosemide was coded as ‘congestive heart failure’ if patients were also on a beta-blocker for congestive heart failure and on ACE inhibitors or sartans.

**Supplementary Table 2.** Prevalence of comorbidity categories included in the RxRisk-V according for First Nations Peoples and non-Indigenous Australians

| **Rx-Risk-V categories*** | **First Nations**  **Peoples** | **Non-Indigenous**  **Australians** |  |
| --- | --- | --- | --- |
|  | **N=89** | **N=3,206** | **p-value** ¥ |
| **Alcohol dependence** | 3 (3.4%) | 58 (1.9%) | 0.24 |
| **Allergies** | 0 (0.0%) | 2 (0.1%) | 1.00 |
| **Anticoagulation** | 2 (2.3%) | 84 (2.8%) | 1.00 |
| **Antiplatelet agents** | 4 (4.6%) | 74 (2.4%) | 0.28 |
| **Anxiety and tension** | 20 (23.0%) | 650 (21.5%) | 0.73 ‡ |
| **Arrhythmias** | 3 (3.4%) | 30 (1.0%) | 0.063 |
| **Benign prostatic hypertrophy** | 0 (0.0%) | 10 (0.3%) | 1.00 |
| **Bipolar disorders** | 3 (3.4%) | 38 (1.3%) | 0.11 |
| **Congestive heart failure -hypertension** | 3 (3.4%) | 26 (0.9%) | 0.046 |
| **Dementia** | 0 (0.0%) | 1 (0.0%) | 1.00 |
| **Depression** | 36 (41.4%) | 843 (27.8%) | 0.006 ‡ |
| **Diabetes** | 10 (11.5%) | 267 (8.8%) | 0.39 ‡ |
| **Epilepsy** | 4 (4.6%) | 140 (4.6%) | 1.00 |
| **Gastric acid disorder** | 24 (27.6%) | 782 (25.8%) | 0.71 ‡ |
| **Glaucoma** | 0 (0.0%) | 27 (0.9%) | 1.00 |
| **Gout** | 1 (1.1%) | 63 (2.1%) | 1.00 |
| **Hepatitis B** | 0 (0.0%) | 11 (0.4%) | 1.00 |
| **HIV** | 0 (0.0%) | 16 (0.5%) | 1.00 |
| **Hyperlipidaemia** | 5 (5.7%) | 243 (8.0%) | 0.55 |
| **Hypertension** | 10 (11.5%) | 348 (11.5%) | 1.00 ‡ |
| **Hyperthyroidism** | 0 (0.0%) | 8 (0.3%) | 1.00 |
| **Hypothyroidism** | 0 (0.0%) | 96 (3.2%) | 0.11 |
| **Incontinence** | 2 (2.3%) | 17 (0.6%) | 0.097 |
| **Irritable bowel syndrome** | 0 (0.0%) | 20 (0.7%) | 1.00 |
| **Ischaemic heart disease - angina** | 2 (2.3%) | 45 (1.5%) | 0.38 |
| **Ischaemic heart disease - hypertension** | 5 (5.7%) | 291 (9.6%) | 0.27 |
| **Liver failure** | 0 (0.0%) | 44 (1.5%) | 0.63 |
| **Malignancies** | 1 (1.1%) | 43 (1.4%) | 1.00 |
| **Migraine** | 0 (0.0%) | 48 (1.6%) | 0.64 |
| **Osteoporosis/Paget’s** | 1 (1.1%) | 58 (1.9%) | 1.00 |
| **Pain (opioids)** | 37 (42.5%) | 1,052 (34.8%) | 0.13 ‡ |
| **Pain (inflammation)** | 16 (18.4%) | 561 (18.5%) | 0.97 ‡ |
| **Pancreatic insufficiency** | 0 (0.0%) | 7 (0.2%) | 1.00 |
| **Parkinson’s disease** | 0 (0.0%) | 29 (1.0%) | 1.00 |
| **Psoriasis** | 0 (0.0%) | 54 (1.8%) | 0.40 |
| **Psychotic illness** | 23 (26.4%) | 402 (13.3%) | <0.001 ‡ |
| **Pulmonary hypertension** | 0 (0.0%) | 3 (0.1%) | 1.00 |
| **Reactive airways diseases** | 23 (26.4%) | 587 (19.4%) | 0.10 ‡ |
| **Renal disease (end stage)** | 1 (1.1%) | 11 (0.4%) | 0.29 |
| **Smoking cessation** | 17 (19.5%) | 268 (8.9%) | <0.001 ‡ |
| **Steroid-responsive conditions** | 10 (11.5%) | 275 (9.1%) | 0.44 ‡ |
| **Transplant** | 0 (0.0%) | 6 (0.2%) | 1.00 |

Note: Data are presented as n (%). * No patients used medications for hyperkalaemia, malnutrition, or tuberculosis, and HCV medications were not included here as all patients included in the OPERA-C study were treated for HCV; ¥ Fisher's exact test unless specified; ‡ Pearson's chi-squared.

**Supplementary Table 3**. Logistic regression analysis of factors associated with loss to follow-up among First Nations Peoples

|  |  | | **No LTFU** | **LTFU** | **Unadjusted** | **Adjusted **** |
| --- | --- | --- | --- | --- | --- | --- |
|  |  | | **N=64** | **N=25** | **OR (95%CI)** | **OR (95%CI)** |
| **Sociodemographic and clinical characteristics *** | | |  |  |  |  |
| **Age** (mean, SD) |  | | 50.72 (9.30) | 41.52 (10.61) | **0.91 (0.86-0.96) ^a^** | **0.93 (0.87-0.99) ^f^** |
| **Gender** Male (vs. female) | | | 45 (70%) | 17 (68%) | 0.90 (0.33-2.43) | N/S |
| **Socioeconomic status** Q4/Q5 most disadvantaged (vs Q1 most affluent/Q2/Q3) | | | 42 (66%) | 18 (72%) | 1.35 (0.49-3.71) | N/S |
| **Remoteness of residence** Regional/Remote (vs Major city) | | | 28 (44%) | 11 (44%) | 1.01 (0.40-2.56) | N/S |
| **Diabetes** |  | | 16 (25%) | 5 (20%) | 0.75 (0.24-2.33) | N/S |
| **Hepatitis B surface antibody** |  | | 24 (44%) | 9 (39%) | 0.83 (0.31-2.24) | N/S |
| **Hepatitis B core antibody** |  | | 21 (42%) | 5 (24%) | 0.43 (0.14-1.36) | N/S |
| **Prescribed opioid substitute** |  | | 16 (25%) | 5 (21%) | 0.77 (0.25-2.41) | N/S |
| **Current alcohol consumption** | Zero alcohol | | 39 (76%) | 15 (71%) | ref | N/S |
|  | <40g/day | | 10 (20%) | 4 (19%) | 1.04 (0.28-3.83) |  |
|  | >=40g/day | | 2 (4%) | 2 (10%) | 2.60 (0.34-20.16) |  |
| **Cirrhosis** |  | | 28 (44%) | 4 (16%) | **0.24 (0.08-0.80) ^b^** | N/S |
| **Liver fibrosis assessment** |  | |  |  |  |  |
| **FIB-4 score** (median, IQR) ^#^ | | | 1.65 (0.90-3.34) | 0.86 (0.60-1.48) | **0.61 (0.38-0.97) ^c^** | **0.71 (0.50-0.99) ^g^** |
| **Liver stiffness** † (kPa)(median, IQR) | | | 8.3 (5.5-16.0) | 5.9 (5.2-9.2) | 0.91 (0.83-1.01) | N/S |
| **Rx-Risk-V comorbidities** | | |  |  |  |  |
| 0 (no Rx-Risk comorbidity) | | | 10 (16%) | 2 (8%) | ref | ref |
| 1 | | | 12 (19%) | 5 (20%) | 2.08 (0.33-13.15) | 3.63 (0.44-29.90) |
| 2 | | | 10 (16%) | 7 (28%) | 3.50 (0.58-21.16) | 2.08 (0.27-15.90) |
| 3 or more RxRisk comorbidities | | | 30 (48%) | 11 (44%) | 1.83 (0.35-9.72) | 2.01 (0.34-11.94) |
| **RxRisk-V categories** | | |  |  |  |  |
| **Pain (opioids)** | | | 27 (44%) | 10 (40%) | 0.86 (0.34-2.22) | N/S |
| **Depression** | | | 27 (44%) | 9 (36%) | 0.73 (0.28-1.90) | N/S |
| **Gastric acid disorders** | | | 17 (27%) | 7 (28%) | 1.03 (0.37-2.90) | N/S |
| **Psychotic illness** | | | 18 (29%) | 5 (20%) | 0.61 (0.20-1.88) | N/S |
| **Anxiety and tension** | | | 13 (21%) | 7 (28%) | 1.47 (0.50-4.26) | N/S |
| **Reactive airways disease** | | | 19 (31%) | 4 (16%) | 0.43 (0.13-1.43) | N/S |
| **Smoking cessation medication** | | | 15 (24%) | 2 (8%) | 0.27 (0.06-1.29) | N/S |
| **Factors related to HCV assessment and treatment *** | | |  |  |  |  |
| **Genotype** G3 (vs not G3) | | | 19 (30%) | 9 (38%) | 1.39 (0.52-3.72) | N/S |
| **Duration of HCV infection** (years; mean, SD) ¥ | | | 23.15 (12.71) | 15.45 (12.70) | **0.95 (0.91-0.99) ^d^** | N/S |
| **Prior HCV treatment** | |  | 9 (14%) | 0 (0%) | - | N/S |
| **Year of HCV treatment initiation** | | |  |  |  |  |
|  | 2016 | | 30 (47%) | 6 (24%) | ref | Ref |
|  | 2017 | | 17 (27%) | 5 (20%) | 1.47 (0.39-5.55) | 3.13 (0.60-16.49) |
|  | 2018-2019 | | 17 (27%) | 14 (56%) | **4.12 (1.34-12.70) ^e^** | **5.14 (1.23-21.36) ^h^** |
| **Type of service** Secondary centre (vs Tertiary hospital) | | | 21 (33%) | 7 (28%) |  | 0.42 (0.11-1.54) |

Note: Reference category (ref) is null exposure unless specified; N/S variable not selected as a predictor;

* We have excluded factors where prevalence of exposure in First Nations Peoples was < 10%;

** The multivariable model was determined based on the results of bi-variable analysis (variables with p≤ 0.2 in unadjusted analysis), informed by a previous study on LTFU in HCV care population,[16] as well as the clinical relevance of variables. The final model included age, FIB-4 score, number of comorbidities assessed by the RxRisk, Year of HCV treatment initiation, and type of service. Statistical significance was set at alpha=0.05, and all p-values were 2-sided;

# missing data for 4 First Nations Peoples;

† missing data for 29 First Nations Peoples;

¥ missing data for 12 First Nations Peoples;

**^a^**  p=0.001;

**^b^** p=0.019;

**^c^** p=0.038;

**^d^** p=0.020;

**^e^** p=0.014;

**^f^** p=0.026;

**^g^** p=0.047;

**^h^** p=0.025.
